# Identification of novel key regulatory lncRNAs in gastric adenocarcinoma

**DOI:** 10.1186/s12864-022-08578-6

**Published:** 2022-05-07

**Authors:** Houri Razavi, Ali Katanforosh

**Affiliations:** Department of Computer and Data Sciences, Faculty of Mathematical Sciences, Shahid Beheshti University, Tehran, Iran

**Keywords:** Stomach adenocarcinoma, lncRNA, mRNA, Survival analysis, Biomarker

## Abstract

**Background:**

Stomach adenocarcinoma (STAD) is one of the most common and deadly cancers worldwide. Recent evidence has demonstrated that dysregulation of long noncoding RNAs (lncRNA) is associated with different hallmarks of cancer. lncRNAs also were suggested as novel promising biomarkers for cancer diagnosis and prognosis. Despite these previous investigations, the expression pattern, diagnostic role, and hallmark association of lncRNAs in STAD remain unclear.

**Results:**

In this study, The STAD lncRNA-mRNA network was constructed based on RNAs that differentially expressed among tumor and normal samples and had a strong expression correlation with others. The high degree nodes of the network were associated with overall survival. In addition, we found that the hubs’ regulatory roles have previously been confirmed in different types of cancers by literature. For example, the HCG22 hub inhibited cell proliferation and invasion and induced apoptosis in oral squamous cell carcinoma (OSCC) cells. The levels of PCNA, Vimentin, and Bcl2 were decreased and E-cadherin and Bax expression was elevated in OSCC cells after HCG22 overexpression. Additionally, HCG22 overexpression inhibited the Akt, mTOR, and Wnt/β-catenin pathways.

Then lncRNAs were mapped to their related GO terms and cancer hallmarks. Based on these mappings, we predict the hallmarks that might be associated with each lncRNA. Finally, the literature review confirmed our prediction.

Among the 20 lncRNAs of the STAD network, 11 lncRNAs (LINC02560, SOX21-AS1, C5orf66-AS1, HCG22, PGM5-AS1, NALT1, ENSG00000241224.2, TINCR, MIR205HG, HNF4A-AS1, ENSG00000262756) demonstrated expression correlation with overall survival (OS). Based on expression analysis, survival analysis, hallmark associations, and literature review, LINC02560, SOX21-AS1, C5orf66-AS1, HCG22, PGM5-AS1, NALT1, ENSG00000241224.2, TINCR, MIR205HG, HNF4A-AS1 plays a regulatory role in STAD. For example, our prediction of association between C5orf66-AS1 expression dysregulation and “sustaining proliferative signal” and “Activating invasion and metastasis” has been confirmed in STAD, OSCC and cervical cancer. Finally, we developed a lncRNA signature with SOX21-AS1 and LINC02560, which classified patients into high and low-risk subgroups with significantly different survival outcomes. The mortality rate of the high-risk patients was significantly higher compared to the low-risk patients (28/1% vs 60.13).

**Conclusion:**

These findings help in designing more precise and detailed experimental studies to find STAD biomarkers and therapeutic targets.

**Supplementary Information:**

The online version contains supplementary material available at 10.1186/s12864-022-08578-6.

## Introduction

Based on the 2020 global cancer observatory data, STAD is the 4th most deadly cancer, with an estimated 1,089,103 incidents and 768,793 deaths in 2020. Thus, novel and comprehensive research on initiation, development, and treatment of STAD seem essential.

For decades, cancer biology focused on the involvement of protein-coding genes. According to recent discoveries, an entire class of molecules, termed non-coding RNA (ncRNA), demonstrates essential regulatory roles in shaping cellular activity. Important examples of this group are coding lncRNAs [1].

Evidence accumulated over the past decade reveals an abundance of lncRNAs interspersed with the coding genes [2, 3]. Whereas the functions of most lncRNAs remain unknown, growing evidence suggests that lncRNAs can modulate chromatin function, regulate the function of membraneless nuclear bodies, change the stability and translation of cytoplasmic mRNAs and interfere with signaling pathways [4]. mRNAs stability modulate by lncRNAs via direct interaction with miRNA or RBP binding sites in target mRNA, detention of miRNAs or RBPs to avoid their interaction with mRNA molecules, enhancing RBP-mRNA interactions by acting as scaffolds and interacting with m6A machinery to modulate m6A levels of target mRNAs [5]. Many of these functions eventually affect gene expression in neuronal disorders, immune responses and cancer [4]. lncRNAs mediate oncogenic or tumor-suppressing effects. Tissue-specific and condition-specific expression patterns suggest that lncRNAs are potential biomarkers and may constitute a new class of cancer therapeutic targets [4, 6, 7].

LncRNAs have vital roles in gene regulation and affect various aspects of cellular homeostasis, including proliferation, invasion, metastasis, apoptosis, migration, or genomic stability [8]. For example, Hong et al. illustrated that lncRNA H19 promotes cell proliferation and invasion by acting as a competing endogenous RNA of miR-138 and releasing EZH2 in oral squamous cell carcinoma, and suggest that H19 may represent a potential therapeutic target for oral squamous cell carcinoma [9]. Ma et al. reported that lncRNA LINC00675 regulates cell proliferation, migration, and invasion by affecting the Wnt/β-catenin signaling pathway in cervical cancer [10]. lncRNA FAL1 functions as an oncogene in hepatocellular carcinoma and could be a diagnostic biomarker or a therapeutic target [11]. lncRNA ANCR is an essential player in breast cancer progression and metastasis through decreasing EZH2 stability. Knockdown of ANCR promoted cell migration and invasion in MCF10A (epithelial cells), whereas ectopic expression of ANCR repressed breast cancer cells migration and invasion [12]. Despite the expanding research on lncRNAs in various cancer, yet we don’t have enough knowledge about key regulatory lncRNAs of STAD.

Typically, differential expression analysis identifies biomarker candidates with significant changes in their expression between normal and disease groups. One drawback of the analysis is that it only considers the changes on single biomolecules like lncRNAs or mRNAs. In differential network analysis, the network typically is built based on correlation, candidate biomarkers select by investigating the network topology. However, correlation tends to generate over-complicated networks, the selection of biomarker candidates purely based on network topology ignores the changes on a single biomolecule level [13]. The assumption is that biomolecules with strongly altered connectivity between distinct biological groups might play a vital role in the disease under study [14]. Differential expression and differential network analysis investigate data from two separate but complementary perspectives: the former focuses on the change of a single biomolecule while the latter concentrates on the change in pairwise association for a biomolecular pair. In this paper, we applied a combination of two models to gain both their advantages.

First, the STAD mRNA-lncRNA network was constructed based on differentially expressed RNAs among normal and tumor samples. Then the most expression correlated RNAs were selected to identify STAD-related lncRNAs. We investigated the hypothesis that hub lncRNAs might have an essential role in STAD. The connection between lncRNAs and cancer hallmarks was identified by connecting mRNAs to their related GO terms and cancer hallmarks. The results prove that hubs of STAD network were cancer-related lncRNAs and could be introduced as biomarkers or therapeutic targets in STAD.

## Material and methods

### Data collection

The mRNA expression profiles of solid tissues for stomach adenocarcinoma (STAD) were downloaded from The Cancer Genome Atlas (TCGA, https://portal.gdc.cancer.gov) using TCGABiolinks package [15]. For mRNA expression data, gene expression was measured through mapping RNA-Seq by Expectation Maximization (RSEM). Initial data consists of 19,947 mRNA from 450 samples where 415 were tumor tissues and 35 were adjacent non-cancerous tissues. We first removed genes with RSEM expression values of 0 in all samples and then log 2 -transformed the expression levels. STAD lncRNAs expression were directly obtained from ‘The Atlas of Noncoding RNAs in Cancer’ (TANRIC, https://ibl.mdanderson.org/tanric/_design/basic/download.html) [16]. In total, 12,727 lncRNAs were quantified for the expression levels as reads per kilo base per million mapped reads (RPKM). Finally, 314 samples, 31 normal and 283 tumors, with both lncRNA and mRNA expression profiles were selected for analysis. lncRNAs were displayed by their ensemble id in TANRIC, so the Ensemble database was used to convert the ensemble ids to gene symbols. Some lncRNAs present by their ensemble id since they were novel transcripts and don’t have a gene symbol.

### Normalization and differential expression

Expression profiles were normalized by TCGAanalyze_Normalization function of TCGAbiolinks. EdgeR package was used to identify the differentially expressed mRNAs and lncRNAs between the normal and tumor samples. 6711 mRNAs and 748 lncRNAs were differentially expressed among two groups. Most significant RNAs with FDR < 0.05 and |logFC| > 2 (424 mRNA and 42 lncRNA) were selected as initial nodes of network.

### Construction of STAD lncRNA-mRNA network

Network-based approaches help us gain a better understanding of the complexities of disease biology. It enables the integration of multiple data and interpreting results at a systems level to allow the understanding of relations among different players involved and corporative functions and regulatory mechanisms. They help to identify potential biomarkers and allow us to characterize networks and sub-networks of biomolecular interactions critical for the emergence of a disease state. A network provides a natural representation of a complex cellular system in which nodes represent the molecular components (RNAs in this work) and edges represent the biological relationships among these entities.

Since, the function of most lncRNAs is unknown, a number of studies have shown that lncRNAs mainly carry out their functions via expression-correlated genes. Here, we tried to carry out the role of critical lncRNAs in STAD by means of a network-based approach. Our hypothesis is that hub RNAs, which have significant differential expression among tumor and normal samples and have strong expression correlation with considerable number of RNAs, could have vital regulatory role in cancer progression and treatment and introduce as cancer biomarkers.

Initial mRNA-lncRNA network of STAD was created by the differentially expressed mRNAs and lncRNAs as initial nodes. Those mRNAs and lncRNAs that were differentially expressed among STAD tumor and normal samples with *P*-value < 0.05 and |logFC| > 2 were selected as initial nodes of the network. Then an edge was defined between lncRNA_A and lncRNA_B, if their expression had changed by a similar function in different situations. In other words, there was an edge between lncRNA_A and lncRNA_B, if they had presented similar alterations in all samples at different stages of STAD. For this purpose, we computed the Pearson correlation coefficient (R) of each RNA pair. All RNA pairs with R > 0.85 and P-value < 0.05 were selected as final edges. For each RNA in a network, degree is defined as the number of edges incident to it. Finally, nodes with zero degrees were omitted from the network. The final STAD network consists of 20 lncRNAs and 88 mRNAs.

### Network visualization

The STAD networks were visualized by Cytoscape 3.8.2, and topology analysis was performed by the package of ‘igraph’ in R language.

### Survival analysis to detect the STAD-specific prognostic signatures

Kaplan–Meier and log-rank tests were used to determine the association between the lncRNAs expression of the STAD network and the overall survival (OS). The samples were labeled based on lncRNAs expression with “Low count” and “High count” according to the threshold defined by *surv_cutpoint* function of *survminer* package. Statistical significance was set at *p*-value < 0.05.

To build a prognostic lncRNA signature to predict survival outcome in STAD, All the 20 lncRNAs were fitted into a multivariate Cox proportional hazards regression model. Risk score was defined based on the Cox regression coefficients of lncRNAs with *p*-value< 0.05 as follows:

$$Risk\ score=\sum_{i=1}^n{\mathit{\exp}}_{lncRNA\_i}\ast {\beta}_{lncRNA\_i}$$

Where “exp” denotes the expression level of lncRNAs, “β” is the cox regression coefficient, and “n” is the number of lncRNAs with Cox regression p-value < 0.05. *surv_cutpoint* function of *survminer* package used to define the threshold to classify the patients into the high- and low-risk groups. Kaplan-Meier survival analyses were carried out to assess the difference in OS between high-risk group and low-risk group, and statistical significance was evaluated using the log-rank test using the R package “survival”.

### STAD lncRNAs associated with cancer hallmarks

To investigate the function and effect of top lncRNAs in the STAD network, first the mRNAs mapped to their Gene Ontology (GO) terms of their biological process using DAVID Bioinformatics Resources (http://david.abcc.ncifcrf.gov). Then the GO terms should map to cancer hallmarks. Chen et al. [17] wrote a review and take together the studies that had mapped GO terms to cancer hallmarks. We used the union of all connections of their reviewed studies that mapped GO terms to cancer hallmarks, for connecting the STAD GO terms to cancer hallmarks. Finally, we construct a flowchart of connections among lncRNAs, mRNAs, GO terms and cancer hallmarks for each module of STAD.

### Literature review

**Fig. 1:**
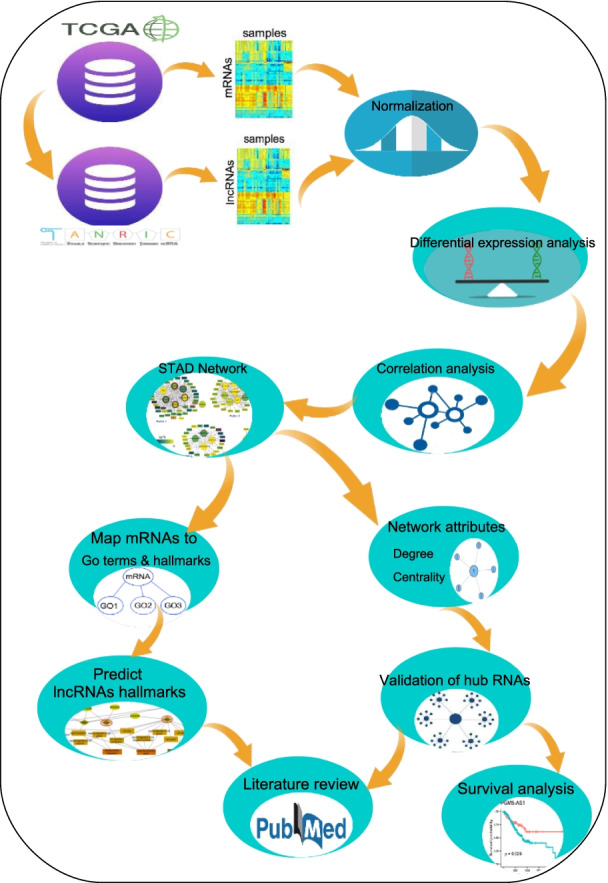
Workflow for key regulatory lncRNA detection in STAD

We used Cosmic cancer gene census to detect oncogenes, tumor suppressor and fusion mRNAs of STAD network. We also applied PubMed search engine to review the related literature to each significant lncRNA. The complete flowchart of paper represents in Fig. 1.

## Results

### Identification of differential expressed RNAs

**Fig. 2:**
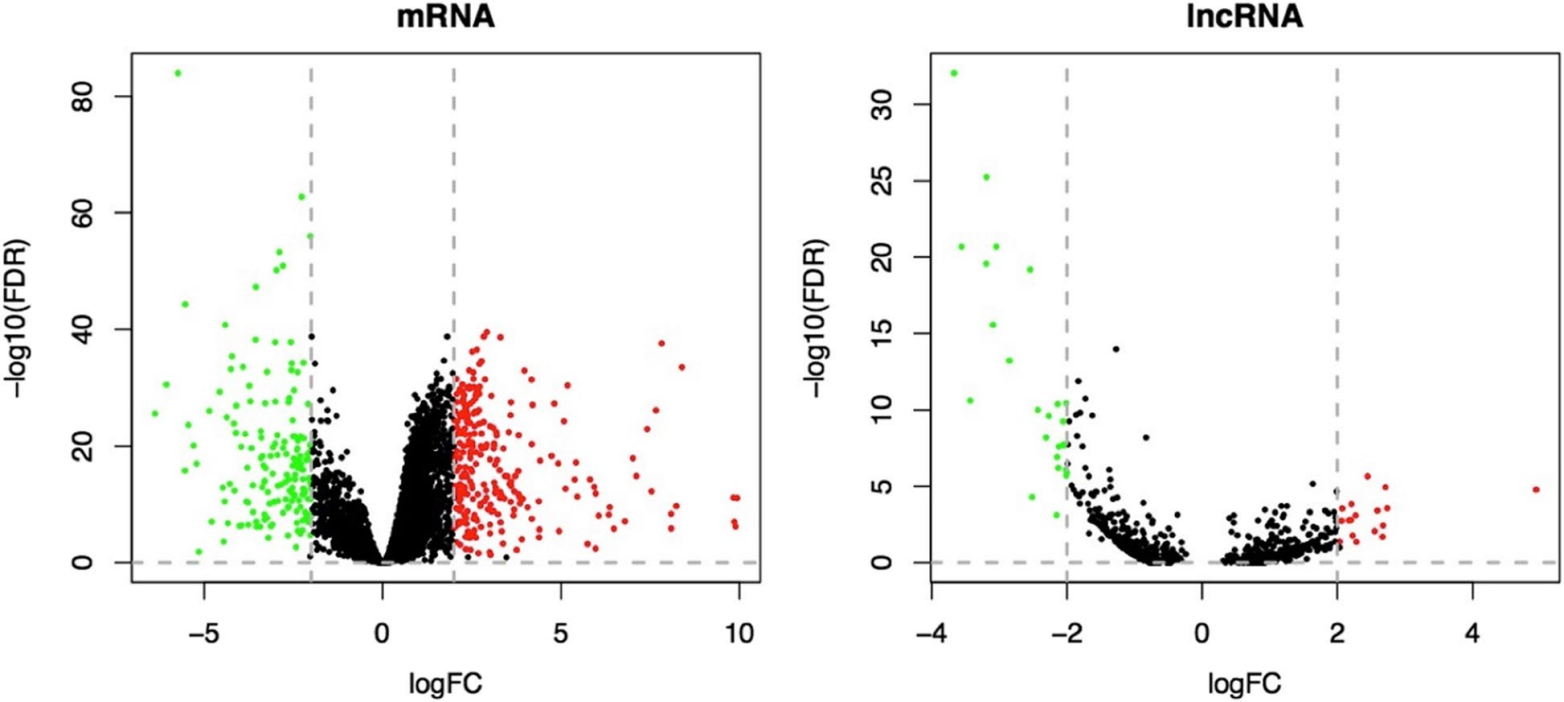
Volcano plots showing the differential expression of mRNAs and lncRNAs in STAD; X-axis indicates the expression differences of mRNAs, and lncRNAs between STAD and normal samples, and Y-axis represents log transformed false discovery rate (FDR) values. Red dots represent the up-regulation and green dots represent down-regulation

We identified the differentially expressed mRNAs and lncRNs in STAD, with *P* < 0.05 and |logFC| > 2 as the thresholds (Supplementary File 1, 2). A total of 424 differential expressed mRNAs (266 up- and 158 down-regulated) and 42 lncRNAs (17 up-, and 25 down-regulated) were detected between STAD and normal samples. Volcano plots displaying the distribution of the mRNAs, and lncRNAs were generated, as shown in Fig. 2.

### STAD lncRNA-mRNA network

**Fig. 3:**
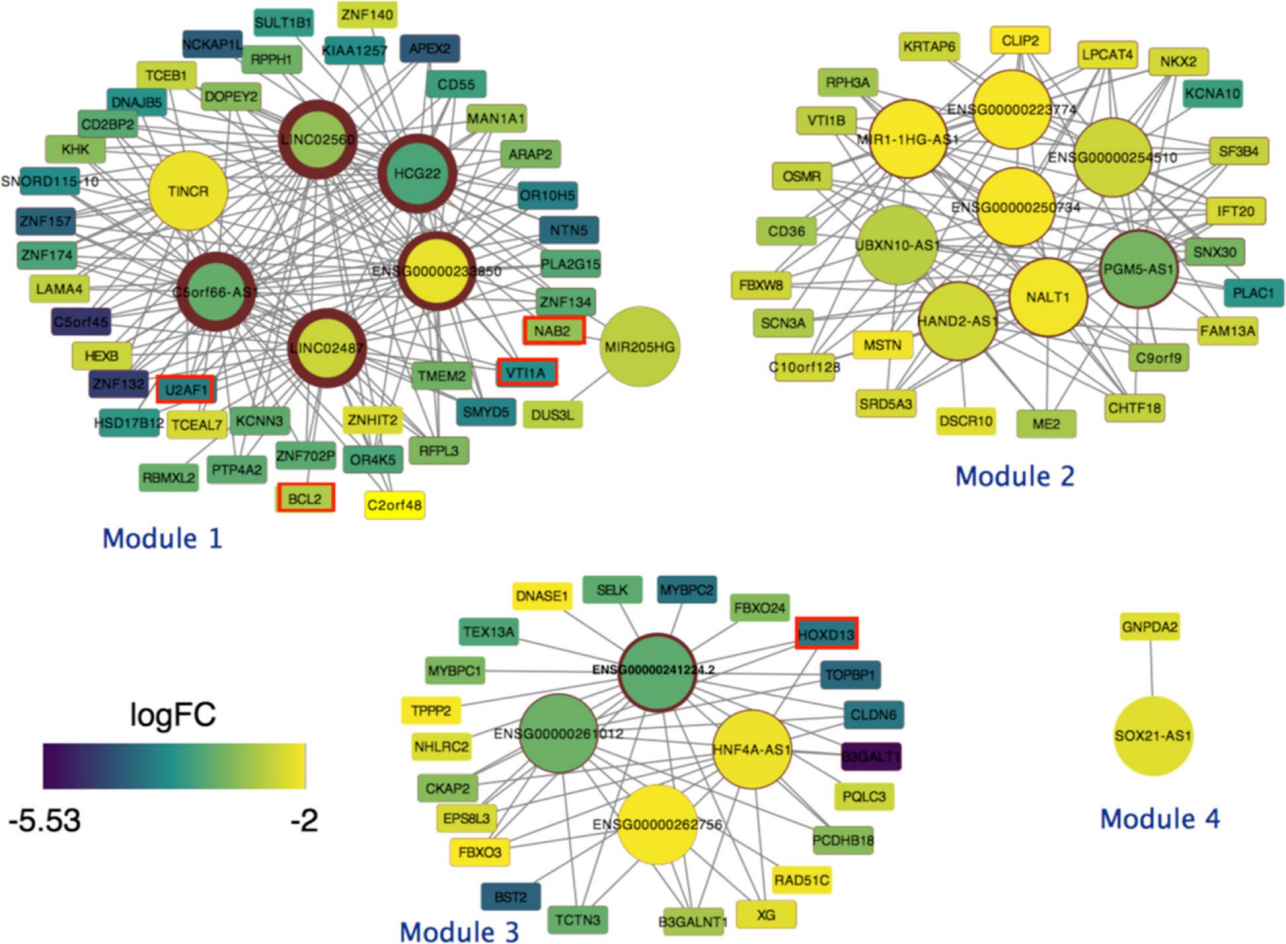
STAD mRNA-lncRNA network. mRNAs represent by rectangles, and lncRNAs represent by circles. Node filling color indicates log fold change of differential analysis among tumor and normal samples. The node’s border thickness represents the node degree

Differentially expressed lncRNAs and mRNAs were considered as initial nodes of the STAD lncRNA-mRNA network (Supplementary File 3). The network’s edges were defined based on Pearson correlation (Supplementary File 4). The final network formed four distinct modules (Fig. 3). Nodes of each module could be related to similar biological processes or the same cancer hallmarks. We will follow this question during the rest of paper. According to network analyses policies, those nodes with the highest degree and betweenness centrality might be more effective in diagnosis and progression of STAD, this will also follow during the rest of paper. Two nodes of module 1,” U2AF1” and “BCL1”, are oncogenes and “NAB2” is a tumor suppressor and “VTI1A” is a fusion gene. In module 3 also “HOXD13” is an oncogene. This evidence and the existence of most high degree nodes in module 1 declare the high importance of this module.

### Correlation between STAD-specific lncRNAs and overall survival

**Fig. 4:**
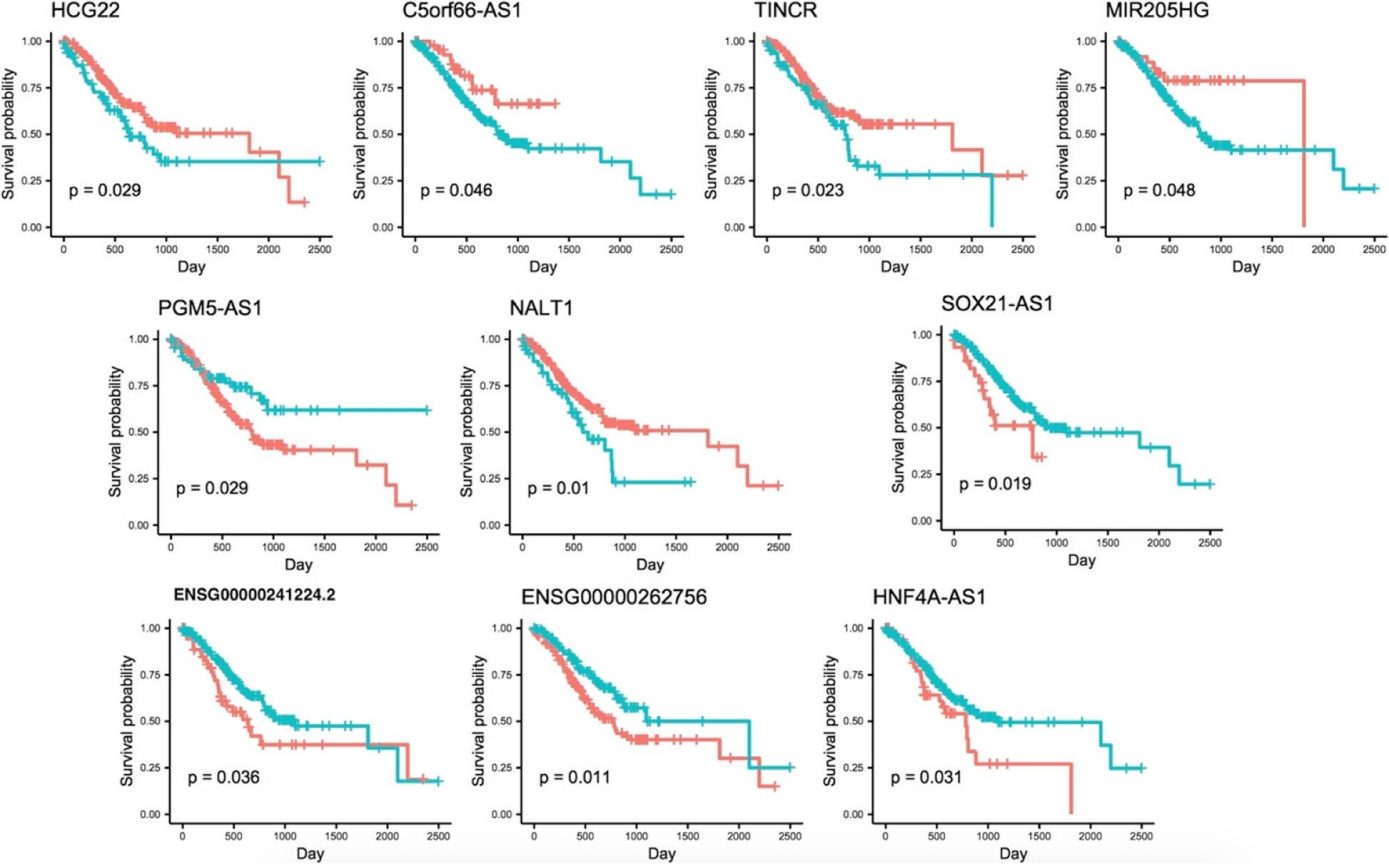
Kaplan–Meier survival curves for significant lncRNAs associated with overall survival of the STAD patients. Log-rank method was used to assess the survival differences between the two groups. Horizontal axis is OS time (days) and vertical axis stands for survival function. The turquoise lines represent the group with low count, and the red lines represent the group with high count

To explore whether the expression of each lncRNA in the STAD network had prognostic significance for predicting overall survival, Kaplan–Meier and log-rank test was used to determine the association between the STAD network lncRNAs and OS. Statistical significance was set at *p*-value < 0.05. Finally, ten lncRNAs, SOX21-AS1, HCG22, C5orf66-AS1, TINCR, MIR205HG, PGM5-AS1, NALT1, ENSG00000241224.2, ENSG00000262756, and HNF4A-AS1 were found to be related to OS (Fig. 4). These ten lncRNAs could serve as prognostic biomarkers of STAD.

### Topological features of the STAD networks

**Fig. 5:**
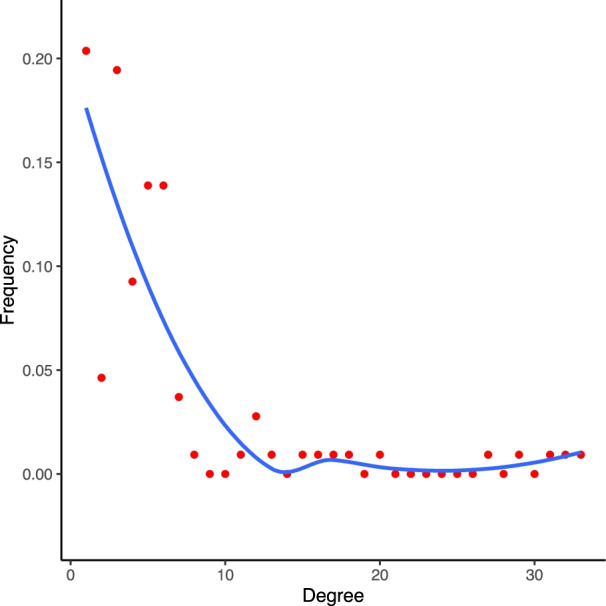
Degree distribution of STAD mRNA-lncRNA network

The STAD mRNA-lncRNA network includes 108 nodes with 88 mRNA and 20 lncRNA which form 4 distinct modules. Based on expression correlation the network consists of 317 edges. As observed, the degree distribution of nodes in STAD mRNA-lncRNA network closely followed a power law distribution (Fig. 5). Most nodes had relatively few interactions with others and only a small portion of nodes had a large number of interactions.

All nodes were sorted in descending order according to their degree and their betweenness centrality (Supplementary File 5).

### STAD lncRNAs signature

**Fig. 6:**
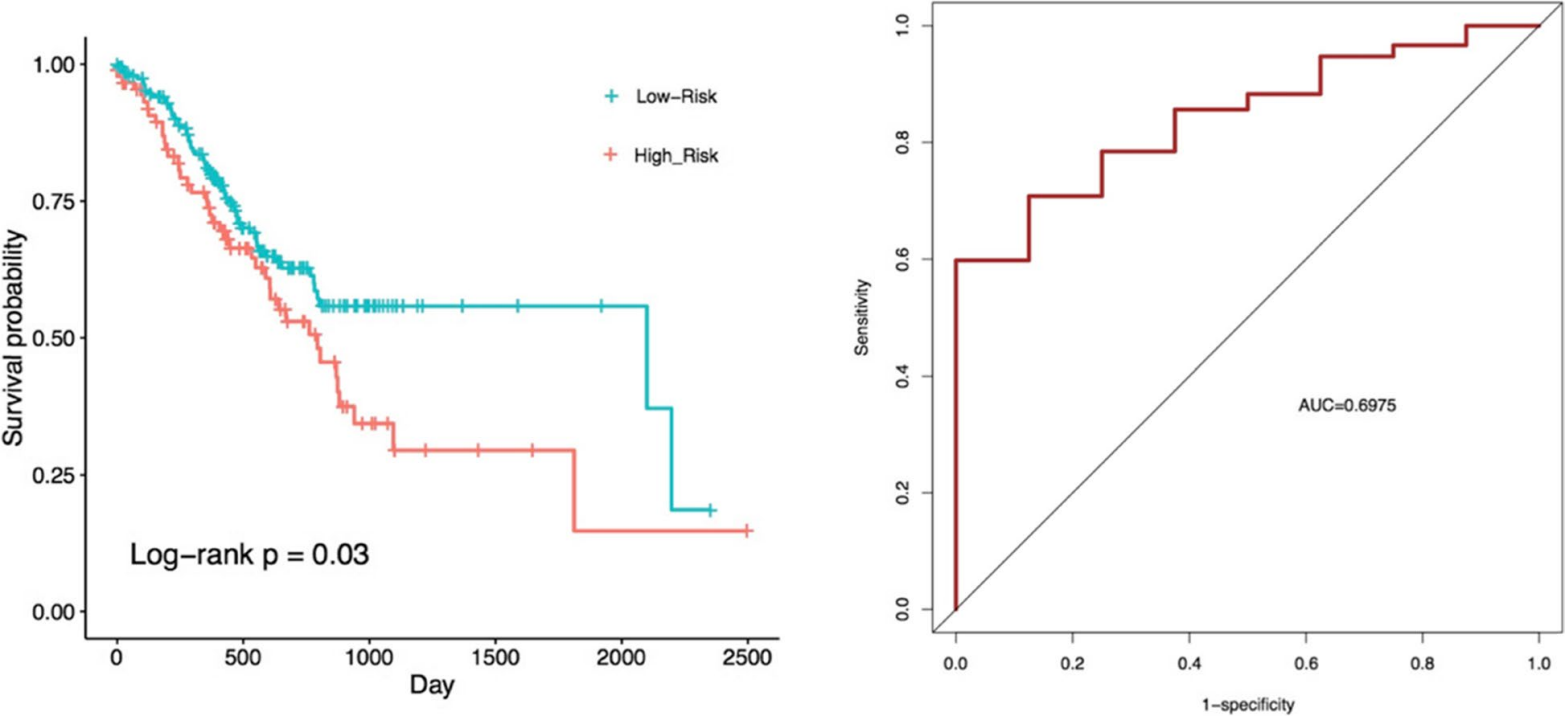
**A.** The survival differences between the high-risk and low-risk groups of lncRNA signature were determined by the log-rank test. **B.** Receiver operating characteristic (ROC) analysis of the risk scores for overall survival prediction

All 20 lncRNAs of STAD network were fitted into the multivariate Cox regression model. The results indicated that only two lncRNAs, SOX21-AS1 and LINC02560, had a significant prognostic value (*p*-value < 0.05) in STAD (Supplementary File 6). Then, these two lncRNAs were used to develop a lncRNA signature. The two-lncRNA signature risk scores were calculated for each patient based on lncRNAs expressions. Finally, the patients were divided into the “Low-risk” (*n* = 153) and “High-risk” (*n* = 153) groups based on their risk scores. The mortality rate of the high-risk patients was significantly higher compared to the low-risk patients (60/13% vs 28/1%). Patients in the high-risk group had significantly shorter survival than those in the low-risk group (Fig. 6). Furthermore, we performed the time-dependent ROC curve analysis to evaluate sensitivity and specificity for survival prediction of two-lncRNA signature. The AUC of STAD signature was 0.6975 (Fig. 6B), demonstrating its utility as a prognostic model for predicting the survival status of STAD.

### Identification of cancer hallmark associated lncRNAs in STAD network

Cancer develops from accumulation of mutations and epigenetic changes. Such events may enforce altered expression of mRNAs. These conversions may contribute to the development and progression of cancer. Therefore, it is essential to explore the interaction between the regulatory systems (such as RNAs) for understanding the function of cells and more complex systems.

**Fig. 7:**
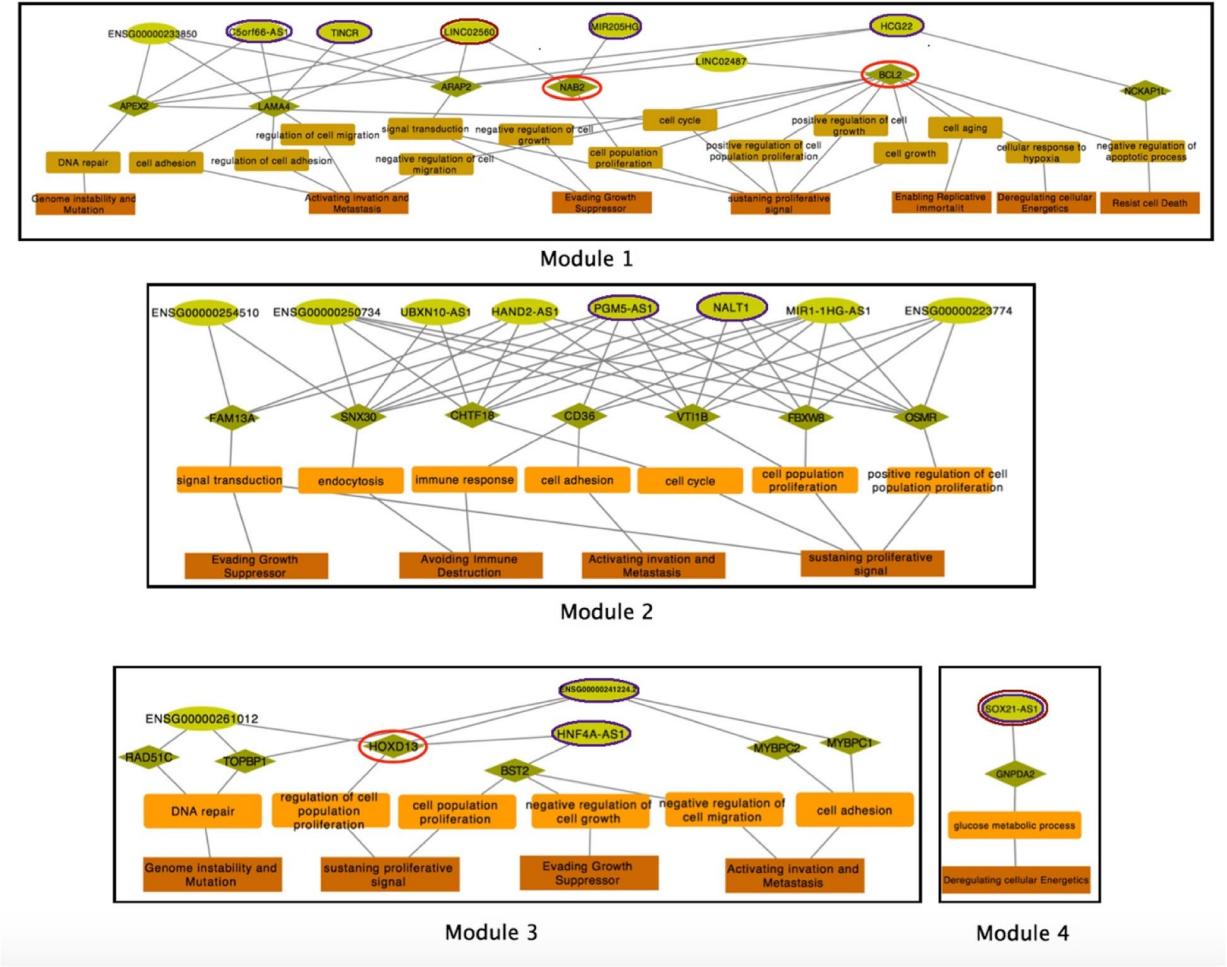
Connection among RNAs and their related GO terms and cancer hallmark. The chart depicts lncRNAs by green circles, mRNAs by green Diamond, GO terms by orange rectangles and cancer hallmarks by brown rectangles. Red circles represent cancer-related genes, blue circles represent lncRNAs that were significantly associated with overall survival and brown circles are those lncRNAs that construct the STAD signature

STAD network was constructed based on differentially expressed RNAs among normal and tumor samples. Then the most expression-correlated RNAs were selected to identify STAD-related lncRNAs. Since there is no annotation for lncRNAs of our STAD network we investigated their function and effects on cancer initiation and progression, via their mRNA neighbors in the network. First, the STAD mRNAs were mapped to their related GO terms. Then, each GO term was mapped to its related cancer hallmarks. Figure 7 represents the results of this mapping. Those RNAs that were not connected to any hallmark were omitted from the network. Since the formation of 4 different modules in the STAD network could be associated with their biological mechanism, we construct the lncRNA-mRNA-hallmark charts for each module separately. Predicted hallmarks of each lncRNAs illustrated in Supplementary File 7.

In Fig. 7, lncRNAs of Module 1 are connected to 7 cancer hallmarks. Generally, hallmarks with higher degrees seem to be more significant than others. “sustaining proliferative signal” has the highest degree among other hallmarks of module one, which is the most fundamental hallmark of cancer. “Activating invasion and metastasis” is the second high-degree hallmark of this module which most leads to death. This could support the high proportion of STAD first module lncRNAs (5/7 = 0.71) that were associated with survival probability among other modules.

LINC02560 (the node with the highest degree, 33, in the STAD network), is connected to “Activating invasion and metastasis “via LAMA4 from three different GO terms which means there could be a strong relationship between LINC02560 and “Activating invasion and metastasis “. On the other hand, the results of cox proportional regression in the previous part demonstrated that the survival probability of patients with downregulation of LINC02560 is twice less than other patients. Furthermore, LINC02560 is connected to “sustaining proliferative signal” via NAB2 and ARAP2, while NAB2 is an oncogene.

The rest of module one lncRNAs are HCG22, C5orf66-AS1, TINCR, and MIR205HG whose expressions were significantly correlated with the patient’s overall survival. Among these, C5orf66-AS1(node with degree 32 in STAD network), and TINCR are also connected to “Activating invasion and metastasis “via three different GO terms from LAMA4.

In module two “sustaining proliferative signal” is also the highest degree and most significant hallmark. PGM5-AS1, NALT1, MIR1-1HG-AS1, and ENSG00000250734 are connected to this hallmark from 4 different paths. The connection between “Avoiding immune destruction” and lncRNAs of module two is a significant distinction among this module and others.

It seems that some lncRNAs of this module could disrupt immune system. Especially, PGM5-AS1 is the only lncRNA of this module that connects to this hallmark via two separate paths. PGM5-AS1 connects to “Avoiding immune destruction” from SNX30 via endocytosis and on the other hand connects to immune response via CD36. Furthermore, we demonstrate that PGM5-AS1 expression is associated with survival probability.

In module three, “sustaining proliferative signal” and “Activating invasion and metastasis” are high-degree hallmarks that both are associated with oncogenesis. HNF4A-AS1 is connected to “sustaining proliferative signal” via HOXD13 and BST2, while HOXD13 is an oncogene and BST2 involves the growth and development of B-cells. This lncRNA expression is also associated with overall survival. ENSG00000241224.2 is another lncRNA of this module that connects to three cancer hallmarks. It is connected to “sustaining proliferative signal” via HOXD13 oncogene.

The fourth module that consists of significant lncRNA, SOX21-AS1, connects to “deregulating cellular energetics” via glucose metabolic process. Malignant tumor cells exhibit fundamentally altered cellular energetics, such as increased aerobic glycolysis, which may contribute to tumorigenesis and malignancy. The connection of SOX21-AS1 to a notable and different cancer hallmark vindicates its separation from the other three STAD modules.

This kind of analysis for defining the connections among lncRNAs, mRNAs, GO terms, and cancer hallmarks could help to have a more precise and comprehensive view of cancer and direct us toward the detection of underlying cancer-related mechanisms. It also directs us in designing new drugs and therapies.

### Validation of STAD key lncRNAs by PubMed literature review

LINC02560 was the highest degree node of the STAD network (degree = 33) and strongly correlated with mortality probability. After fitting the STAD lncRNAs into the multivariate Cox regression model, it had the highest hazard ratio (HR = 2.42) and highest *p*-value (0.004). It indicates that the probability of death in patients with low expression of LINC02560 is 2.42 times higher than others.

It also has a strong expression correlation with U2AF1oncogene, VTI1A fusion gene, and NAB2 tumor suppressor. According to Fig. 7, LINC02560 might be associated with “sustaining proliferative signal” and “Activating invasion and metastasis”. Zhou et al. have reported that dysregulation of LINC02560 expression was significantly associated with overall survival in squamous cell carcinoma of tongue [18].

C5orf66-AS1 is the second high-degree node of the STAD network (degree = 32) and its’ expression was found to be associated with OS. It also has a strong expression correlation with U2AF1oncogene and VTI1A fusion gene. According to Fig. 7, C5orf66-AS1 might be related to “sustaining proliferative signal” and “Activating invasion and metastasis”. We consider the hallmarks with degree> 2 from the specified lncRNA. C5orf66-AS1 is an antisense lncRNA, and growing evidence has implicated that a large number of antisense lncRNAs play crucial roles in cancer [19–21].

Expression levels of C5orf66-AS1 in gastric cancer tissues, serum, and cell lines were used for analysis in [22]. C5orf66-AS1 expression was downregulated in gastric cancer cells compared to that in adjacent normal tissues. Serum C5orf66-AS1 levels were significantly lower in gastric cancer patients than in superficial gastritis and atrophic gastritis patients. Low serum expression of C5orf66-AS1 was associated with an increased risk of gastric dysplasia and gastric cancer. They introduced C5orf66-AS1 a Potential Biomarker for Predicting Early Gastric Cancer [22].

Downregulation and aberrant hypermethylation of C5orf66-AS1 have been detected in limited several tumors. Guo et al. have investigated the expression status and function of C5orf66-AS1 in gastric cardia adenocarcinoma. C5orf66-AS1 was significantly downregulated in GCA tissues and cell lines, and the expression level was associated with TNM stage, pathological differentiation, lymph node metastasis, and distant metastasis or recurrence. C5orf66-AS1 expression was significantly increased in cancer cells after being treated with 5-Aza-dC. Further methylation analysis demonstrated that the aberrant hypermethylation of the regions around the transcription start site of C5orf66-AS1 was more tumor-specific and was associated with its expression. In addition, C5orf66-AS1 inhibited gastric cancer cell proliferation and invasion, and the dysregulation and hypermethylation of the regions around the transcription start site of C5orf66-AS1 were associated with poorer gastric cardia adenocarcinoma patients’ survival [23].

Lu et al. have also demonstrated that C5orf66-AS1 prevents oral squamous cell carcinoma (OSCC) through inhibiting cell growth and metastasis. Their findings revealed that the expression of lncRNA C5orf66-AS1 in OSCC tissues and cells was significantly decreased. Overexpression of C5orf66-AS1 significantly inhibited the proliferation, invasion and migration ability of OSCC cells, and promoted cell apoptosis, while C5orf66-AS1 downregulation presented the opposite effects [24].

Rui et al. have reported that C5orf66-AS1 was significantly upregulated in cervical cancer tissues and cells. Over-expression of C5orf66-AS1 promoted the proliferation of cervical cancer cells, while downregulation of C5orf66-AS1 promoted the apoptosis of cervical cancer cells [25]. Yu et al. have also demonstrated that C5orf66-AS1 is downregulated in pituitary null cell adenomas and is associated with their invasiveness [26]. Sailer et al. have illustrated that knockdown of C5orf66-AS1 significantly inhibits the proliferation, invasion, and migration of U2OS cells in Osteosarcoma, and stimulated cell apoptosis [27].

LINC02487 is the third high-degree node of the STAD network (degree = 31) and also has the highest betweenness centrality among the whole nodes. LINC02487 had a strong expression correlation with oncogenes U2AF1 and BCL2 and VTI1A fusion gene. According to Fig. 6, C5orf66-AS1 might be associated with “sustaining proliferative signal”. Feng et al. have reported tumor Suppressor *LINC02487* downregulation in Oral Squamous Cell Carcinoma. Their functional studies demonstrate that overexpression of *LINC02487* significantly suppresses cell migration and invasion and also inhibits cell proliferation. For the mechanism, they revealed that *LINC02487* directly binds to USP17, a deubiquitinating enzyme, and regulates cell migration and invasion through the USP17-SNAI1 axis in a process that involves epithelial-mesenchymal transition (EMT) [28]. Li et al. also introduced *LINC02487as* Biomarker for Early Diagnosis of Oral Squamous Cell Carcinoma [29].

HCG22 is the fourth high degree node of the STAD network (degree = 29) and also has the second high-centrality among the whole nodes. HCG22 expression was found to be associated with OS. HCG22 had a strong expression correlation with the U2AF1 oncogene and VTI1A fusion gene. According to Fig. 7, HCG22 might be related to “sustaining proliferative signal” and “Activating invasion and metastasis “.

Wang et al. found that HCG22 was weakly expressed in oral squamous cell carcinoma (OSCC) cells. HCG22 overexpression inhibited cell proliferation and invasion and induced apoptosis in OSCC cells. The levels of PCNA, Vimentin, and Bcl2 were decreased and E-cadherin and Bax expression was elevated in OSCC cells after HCG22 overexpression. Additionally, HCG22 overexpression inhibited the Akt, mTOR, and Wnt/β-catenin pathways. Activation of Akt, mTOR and Wnt/β-catenin pathways attenuated the anti-tumor property of HCG22 in OSCC cells. Furthermore, HCG22 overexpression inhibited the growth of OSCC cells in vitro and in vivo. In conclusion, HCG22 exerted anti-tumor properties in OSCC by inhibiting the Akt, mTOR, and Wnt/β-catenin pathways [30]. Cui et al. also introduces HCG22 as a key lncRNA in competing for endogenous RNA network in laryngeal squamous cell carcinoma [31]. Totally, we obtain 28 research in PubMed that introduce HCG22 as a key lncRNA in diverse cancers, but none of them were investigated on STAD.

ENSG00000233850 is the fifth high degree node of STAD network (degree = 27). This lncRNA has a strong expression correlation with the U2AF1 oncogene and VTI1A fusion gene. According to Fig. 7, it might be associated with “Activating invasion and metastasis”. ENSG00000233850 is a novel lncRNA. It is only reported as a downregulated lncRNA in head-and-neck squamous cell carcinoma [32].

TINCR is a low degree lncRNA of the STAD network (degree = 7) but it has a strong expression correlation with oncogene U2AF1. HCG22 expression was found to be related to OS. According to Fig. 7, it might be related to “Activating invasion and metastasis”. Zheng et al. declare that mounting evidence has indicated that TINCR contributes to various cellular processes, such as proliferation, apoptosis, autophagy, migration, invasion, and metastasis [33].

Zheng et al. has reported that lncRNA TINCR was significantly upregulated in nasopharyngeal carcinoma (NPC) and was positively associated with poor survival. TINCR Silencing inhibited NPC progression and cisplatin resistance. TINCR bound ACLY and protected it from ubiquitin degradation to maintain total cellular acetyl-CoA levels. Accumulation of cellular acetyl-CoA promoted de novo lipid biosynthesis and histone H3K27 acetylation, which ultimately regulated the peptidyl arginine deiminase 1 (PADI1)-MAPK-iMMP2/9 pathway. These findings demonstrate that TINCR acts as a crucial driver of NPC progression and chemoresistance [34].

Trastuzumab resistance followed by metastasis is a major obstacle in improving the clinical outcome of patients with advanced HER-2+ breast cancer. Dong et al. prove that TINCR could promote trastuzumab resistance and the accompanied Epithelial-mesenchymal Transition process in breast cancer. Therefore, TINCR might be a potential indicator for prognosis and a therapeutic target to enhance the clinical efficacy of trastuzumab treatment [35]. Furthermore, Wang et al. demonstrated that serum lncRNA TINCR level was significantly increased in breast cancer, especially in triple-negative breast cancer (TNBC). High circulating lncRNA TINCR was significantly correlated with worse clinicopathological features and clinical outcome of TNBC. Finally, they declared that serum lncRNA TINCR might be a useful novel and non-invasive biomarker for the prognosis prediction of TNBC [36].

Xu et al. found that the expression of TINCR was significantly increased in bladder cancer tissues and cell lines. Moreover, the high expression of TINCR was associated with tumor metastasis and advanced tumor, metastasis stage, as well as reduced overall survival rates of patients with bladder cancer. Further investigation revealed that microRNA-7 was negatively mediated by TINCR in bladder cancer cells. Silencing of TINCR expression significantly increased miR-7 expression and reduced bladder cancer cell proliferation, migration, and invasion, while knockdown of miR-7 expression reversed the inhibitory effects of TINCR downregulation on bladder cancer cells [37]. Increasing number of research on TINCR lncRNA in diverse cancer from 2019 declares its impressive role.

MIR205HG is a low degree node in the STAD network, but it has a strong expression correlation with NAB2 tumor suppressor. On other hand, MIR205HG expression dysregulation was found to be associated with OS. According to Fig. 7, it might be related to “sustaining proliferative signal”. MIR205HG acts as a competing endogenous RNA to expedite cell proliferation and progression via targeting miR-299-3p in lung squamous cell carcinoma [38]. MIR205HG was upregulated in cervical cancer tissues and cell lines, and its depletion inhibited the proliferation and metastasis of cervical cancer cells [39]. Long Non-coding MIR205HG Depletes Hsa-miR-590-3p Leading to unrestrained proliferation in Head and Neck Squamous Cell Carcinoma [40]. Song et al. studied the involvement of the *miR205HG* in the development of Barrett’s esophagus (BE) and esophageal adenocarcinoma (EAC) to clarify the role of *miR205HG* in in-vitro and in-vivo experiments. They revealed that *miR205HG* was downregulated in EAC vs. normal esophageal epithelia (NE) as well as in EAC cell lines, and its forced overexpression inhibited EAC cell proliferation and cell cycle progression in vitro. Similarly, overexpression of MIR205HG inhibited xenograft tumor growth in mice in vivo. Finally, their findings declared that MIR205HG functions as a tumor suppressor in the development of BE and EAC, at least in part through its effect on the Hedgehog signaling pathway [41].

Up to here, we investigate the role of module 1 lncRNAs in the literature. In module 2, PGM5-AS1 is the node with the highest degree (degree = 18). This lncRNA expression dysregulation was found to be associated with OS. “sustaining proliferative signal” and “avoiding immune destruction” are the associated high degree hallmarks of this lncRNA.

Du et al. study the inhibitory role of human PGM5-AS1 in the proliferation and apoptosis of prostate cancer (PC) cells. PGM5-AS1 is expressed at low levels in PC cell lines. Forced overexpression of PGM5-AS1 restricted proliferation and facilitated apoptosis of PC cells, manifesting in suppressed xenograft tumor growth in nude mice. They validated that the anti-cancer role of PGM5-AS1 in PC cells was achieved by binding to miR-587 to promote the expression of GDF10 [42].

Zhou et al. found that PGM5-AS1 was low expressed in human colorectal cancer cells, which could promote tumor proliferation, migration, and invasion by serving as a molecular sponge. It modulated the inhibitory effect of miR-100-5p on tumor suppressor gene SMAD4 [43].

According to Qian et al.’s findings, the PGM5-AS1expression in clear cell renal cell carcinoma (CCRCC) specimens was lower than those in matched non-tumors. Downregulation of PGM5-AS1 was closely associated with more advanced clinical features, including lymph nodes metastasis and distant metastasis. A clinical study revealed that CCRCC patients with lower PGM5-AS1 expressions had substantially shorter overall survival (OS) and disease-free survival (DFS) than patients with higher PGM5-AS1 expressions. Further multivariate assays demonstrated that PGM5-AS1 was identified as an independent prognostic factor for patients with CCRCC. Finally, they concluded that Down-regulation of PGM5-AS1 in CCRCC tissues had a strong association with unfavorable outcomes and PGM5-AS1 might be a potential tumor suppressor [44].

NALT1 is the second high-degree node of module 2 of the STAD network (degree = 17). This lncRNA expression dysregulation was found to be associated with OS. According to Fig. 7, it might affect “sustaining proliferative signal”. Yet, no experimental study investigates its role in cancer initiation or progression.

The HAND2-AS1 degree in module 2 is 15. It also holds the highest betweenness centrality among whole nodes of module 2. Da et al. reviewed HAND2- AS1 related studies in late 2020. They reported that HAND2- AS1 is downregulated in common tumors such as hepatocellular carcinoma (HCC) and colorectal cancer (CRC). Low expression of HAND2-AS1 is associated with the early development and prognosis of many types of tumors. They believe that it can act as a prognostic marker and therapeutic target for many kinds of tumors. Regulation of HAND2-AS1 in numerous tumor systems can affect the proliferation and other phenotypes of cancer cells, such as osteosarcoma, esophageal squamous cell carcinoma, endometrioid carcinoma, and colorectal cancer [45].

MIR1-1HG-AS1, ENSG00000250734, ENSG00000223774 are other lncRNAs of module 2 that were differentially expressed among tumor and normal samples and had strong correlations with module 2 lncRNAs. These lncRNAs are novel and we hadn’t found their related study in the literature. According to Fig. 7, we believe that they might be associated with “sustaining proliferative signal”.

In module 3, lncRNA ENSG00000241224.2 is the sixth high-degree node of the STAD network (degree = 6) and the first high-degree node in the module. It is also the fourth high-betweenness node of the STAD network. This lncRNA expression dysregulation was found to be associated with OS. Though it is a novel lncRNA, it has a strong correlation expression with HOXD13 oncogene. Although there is no evidence about the role of ENSG00000241224.2 on cancer, its’ probable relationships with other mRNAs, GO terms, and cancer hallmarks are illustrated in Fig. 7.

HNF4A-AS1 is another lncRNA of module 3 that has a strong correlation expression with HOXD13 oncogene and its expression dysregulation was found to be associated with OS. HNF4A-AS1 possible relationship with other mRNAs, GO terms, and cancer hallmarks illustrated in Fig. 7. HNF4A-AS1 is introduced as a prognostic biomarker in lung adenocarcinoma and hepatocellular carcinoma [46, 47]. Song et al. also suggested that therapeutic targeting of HNF4A-AS1/hnRNPU/CTCF axis inhibits aerobic glycolysis and neuroblastoma progression [48].

SOX21-AS1 is the only lncRNA of module 4 and its loneliness could be associated with it’ distinct function. SOX21-AS1 expression dysregulation was found to be associated with OS. This significant lncRNA was selected for STAD lncRNA signature in previous parts. According to Fig. 7, SOX21-AS1 might associate with “deregulating cellular energetics”. Lack of sufficient predefined relations among GO terms and cancer hallmarks, leads to limited number of connections among this lncRNA and cancer hallmarks. Literature review declares that LncRNA SOX21-AS1 is dysregulated in many types of human cancers, and functions as a tumor suppressor or promoter depending on tumor types [49].

Gai et al.’s findings illustrated SOX21-AS1 upregulation in glioma tissues and cells. In vitro, SOX21-AS1 knockdown repressed proliferation, migration, invasion, and enhanced apoptosis in glioma cells. In vivo, SOX21-AS1 knockdown suppressed tumor growth in mice. Their results indicated that SOX21-AS1/miR-144-3p/PAK7 axis played an oncogenic role in glioma cells by regulating the Wnt/β-catenin pathway, which suggests a rational therapeutic strategy for glioma [50]. To the best of our knowledge, yet no experimental study investigates the SOX21-AS1 role in STAD, but our results confirm its key regulatory role in STAD.

This comprehensive literature review of significant lncRNAs of the STAD network provides a comprehensive view that could help to investigate STAD biomarkers and therapeutic targets.

## Discussion

Differential expression analysis is frequently used to identify biomarker candidates with significant changes in their expression levels between distinct biological groups. One drawback of the analysis is that it only considers the changes on a single biomolecule level. Recently, differential network analysis has become popular due to its capability to measure the changes on the biomolecular pair level. In differential network analysis, the network is typically built based on correlation, and biomarker candidates are selected by investigating the network topology. However, correlation tends to generate over-complicated networks and the selection of biomarker candidates purely based on network topology ignores the changes on a single biomolecule level. In this paper, we propose a novel mixture model. First, we detected RNAs that were differentially expressed among tumor and normal samples, then the correlation among these RNAs was computed. Finally, the STAD network we constructed by RNAs that were differentially expressed among tumor and normal samples and had a high correlation with other RNAs of networks. This leads to the construction of a sparse network with 20 lncRNAs and 88 mRNAs that help us in the further analysis.

We also try to discover the roles of STAD network lncRNAs. First, we mapped the lncRNAs to their associated GO terms. Chen et al. [17] had a review on the whole four studies that mapped the GO terms to cancer hallmarks. Go-term mapping was done based on their study. The literature review proved the accuracy of the results.

Since their mapping was associated with a limited number of GO terms, this leads to the absence of U2AF1 oncogene and VTI1A in the lncRNA-mRNA-GO term- hallmark network. By extension of such studies and definition of more mappings, we would have a better understanding and discoveries.

Furthermore, although some lncRNAs of the STAD network, like ENSG00000241224.2, are novel transcripts, their differential expression among tumor and normal samples and their strong correlation with lots of mRNAs and their expression association with OS prove their regulatory role in STAD.

## Conclusion

Frontier evidence suggests that dysregulation of lncRNA is ubiquitous in all human tumors. lncRNAs contribute to various cellular processes, such as proliferation, invasion, metastasis and apoptosis, but yet their molecular mechanisms in STAD have not been fully studied. Therefore, the current study comprehensively investigated the regulatory role of lncRNAs in STAD. The STAD lncRNA-mRNA network was constructed based on RNAs that were differentially expressed among tumor and normal samples and had a strong expression correlation with others. Our results proved that high degree nodes of this network have an association with overall survival and their regulatory roles have been confirmed in diverse cancer.

Then mRNAs were connected to their GO terms. To predict the associated hallmark of each lncRNA, we applied previous research that proved the association between some mRNAs and cancer hallmarks. As a consequence, we predicted the hallmarks that might be associated with each lncRNA. Finally, the literature review confirmed our prediction. Our study provides a comprehensive perspective of STAD lncRNA signature that could help us toward more detailed and precise computational and experimental studies.

## Supplementary Information

**Additional file 1: Supplementary File 1.** List of mRNAs that were differentially expressed among normal and tumor samples. **Supplementary File 2.** List of lncRNAs that were differentially expressed among normal and tumor samples. **Supplementary File 3.** Nodes of the STAD lncRNA-mRNA network. **Supplementary File 4.** Edges of the STAD lncRNA-mRNA network with their correlations and *p*-values. **Supplementary File 5.** Hub nodes attributes. **Supplementary File 6.** Cox Regression Results. **Supplementary File 7.** Related hallmarks of hub nodes.

## Data Availability

The mRNA expression profiles were downloaded from TCGA (https://portal.gdc.cancer.gov) and lncRNAs expression were directly obtained from ‘The Atlas of Noncoding RNAs in Cancer’ (TANRIC, https://ibl.mdanderson.org/tanric/_design/basic/download.html). The rest of the information included within the article and its additional files.

## Abbreviations

STAD: Stomach Adenocarcinoma
lncRNA: Long non-coding RNA
ncRNA: Non-coding RNA
TCGA: The Cancer Genome Atlas
RSEM: RNA-Seq by Expectation Maximization
TANRIC: The Atlas of Noncoding RNAs in Cancer
RPKM: Reads per kilo base per million mapped reads
OS: Overall Survival
GO: Gene Ontology
OSCC: Oral Squamous Cell Carcinoma
EMT: Epithelial-mesenchymal Transition
NPC: Nasopharyngeal Carcinoma
PADI1: Peptidyl Arginine Deiminase 1
TNBC: Triple-negative Breast Cancer
BE: Barrett’s Esophagus
EAC: Esophageal Adenocarcinoma
NE: Esophageal epithelia
PC: Prostate Cancer
DFS: Disease-free Survival
HCC: Hepatocellular Carcinoma
CCRCC: Clear Cell Renal Cell Carcinoma
