# Supplementary material for: Identification of novel key regulatory lncRNAs in gastric adenocarcinoma
Source: BMC Genomics. 2022 May 7;23:352. doi: 10.1186/s12864-022-08578-6 (PMC9080188; doi:10.1186/s12864-022-08578-6)
Supplement: Supplementary file 1 — Additional file 1: Supplementary File 1. List of mRNAs that were differentially expressed among normal and tumor samples. Supplementary File 2. List of lncRNAs that were differentially expressed among normal and tumor samples. Supplementary File 3. Nodes of the STAD lncRNA-mRNA network. Supplementary File 4. Edges of the STAD lncRNA-mRNA network with their correlations and p-values. Supplementary File 5. Hub nodes attributes. Supplementary File 6. Cox Regression Results. Supplementary File 7. Related hallmarks of hub nodes. [file 12864_2022_8578_MOESM1_ESM.zip › Supplementary 1/sup6_cox regression.docx]

Supplementary 6:

**Table 1: Cox regression coefficient**

| lncRNA | cox regression coef | | exp(coef) | pvalue |
| --- | --- | --- | --- | --- |
| LINC02487 | -0.11 | 0.89 | | 0.76 |
| HNF4A-AS1 | -0.17 | 0.91 | | 0.72 |
| MIR1-1HG-AS1 | 1.29 | 3.65 | | 0.18 |
| ENSG00000223774 | -0.53 | 0.58 | | 0.36 |
| PGM5-AS1 | 0.09 | 1.09 | | 0.45 |
| HCG22 | -0.08 | 0.91 | | 0.72 |
| ENSG00000233850 | -1.81 | 0.16 | | 0.27 |
| C5orf66-AS1 | -0.08 | 0.92 | | 0.62 |
| ENSG00000241224.2 | 0.28 | 1.33 | | 0.16 |
| HAND2-AS1 | 0.03 | 1.03 | | 0.85 |
| NALT1 | -0.68 | 0.5 | | 0.31 |
| ENSG00000250734 | -0.33 | 0.71 | | 0.22 |
| LINC02560 | 0.88 | 2.42 | | 0.004 |
| ENSG00000261012 | -0.11 | 0.89 | | 0.72 |
| ENSG00000254510 | 0.04 | 1.04 | | 0.96 |
| TINCR | -0.15 | 0.85 | | 0.43 |
| UBXN10-AS1 | 0.16 | 1.17 | | 0.37 |
| MIR205HG | 0.05 | 0.95 | | 0.61 |
| ENSG00000262756 | -0.06 | 0.93 | | 0.93 |
| SOX21-AS1 | 0.14 | 1.15 | | 0.04 |
